# Yellow Dioxobilin‐Type Tetrapyrroles from Chlorophyll Breakdown in Higher Plants—A New Class of Colored Phyllobilins

**DOI:** 10.1002/chem.201806038

**Published:** 2019-02-19

**Authors:** Chengjie Li, Theresia Erhart, Xiujun Liu, Bernhard Kräutler

**Affiliations:** ^1^ Institute of Organic Chemistry & Centre of, Molecular Biosciences Innsbruck (CMBI) University of Innsbruck Innrain 80/82 6020 Innsbruck Austria; ^2^ Present address: Key Laboratory for Advanced Materials and Institute of Fine Chemicals, School of Chemistry & Molecular Engineering East China University of Science & Technology Meilong Rd 130 200237 Shanghai China; ^3^ Present address: Research Center of Analysis and Test East China University of Science & Technology Meilong Rd 130 200237 Shanghai China

**Keywords:** green chemistry, heterocycles, photoisomerization, phyllobilin, porphyrinoids

## Abstract

In senescent leaves chlorophyll (Chl) catabolites typically accumulate as colorless tetrapyrroles, classified as formyloxobilin‐type (or type‐I) or dioxobilin‐type (type‐II) phyllobilins (PBs). Yellow type‐I Chl catabolites (YCCs) also occur in some senescent leaves, in which they are generated by oxidation of colorless type‐I PBs. A yellow type‐II PB was recently proposed to occur in extracts of fall leaves of grapevine (*Vitis vinifera*), tentatively identified by its mass and UV/Vis absorption characteristics. Here, the first synthesis of a yellow type‐II Chl catabolite (DYCC) from its presumed natural colorless type‐II precursor is reported. A homogenate of a *Spatiphyllum wallisii* leaf was used as “green” means of effective and selective oxidation. The synthetic DYCC was fully characterized and identified with the yellow grapevine leaf pigment. As related yellow type‐I PBs do, the DYCC functions as a reversible photoswitch by undergoing selective photo‐induced *Z*/*E* isomerization of its C15=C16 bond.

The seasonal disappearance of chlorophyll (Chl) and the concomitant development of the fall colors have been a fascinating puzzle, unsolved until about 30 years ago.^1^ As we know now, senescence‐related breakdown of Chl occurs in a strictly regulated way in higher plants^2^ and produces abundant bilin‐type Chl catabolites, named phyllobilins (PBs).^3^ Indeed, a range of colorless typical PBs are generated along the common “PAO/phyllobilin” pathway2a, 4 (PAO=pheophorbide a oxygenase) and accumulate in senescent leaves.^5^ They are natural descendants of the “red” Chl catabolite (RCC),2b, 6 a cryptic red 1‐formyl‐19‐oxobilin that originates from the specific oxidative cleavage of the macrocycle of pheophorbide *a* by PAO (see Scheme 1).^7^ Efficient enzymatic reduction of the C15=C16 bond RCC^8^ produces colorless, blue fluorescent formyloxobilins (fluorescent type‐I PBs), named “primary” fluorescent Chl catabolites (*p*FCCs).^9^ Typical modified fluorescent Chl catabolites (FCCs) are enzymatically produced2b from *p*FCCs and isomerize spontaneously in the weakly acidic medium of the vacuoles, to corresponding “nonfluorescent” Chl catabolites (NCCs).^10^ In *Arabidopsis thaliana*, *p*FCCs and the corresponding 3^2^‐hydroxy‐*p*FCCs are deformylated effectively by the cytochrome‐P450‐type oxygenase CYP89A9, opening up a path to fleetingly existent dioxobilin‐type fluorescent Chl catabolites (DFCCs),^11^ such as *At*‐DFCC‐33,^12^ and to their abundant nonfluorescent isomers, such as *At*‐DNCC‐33, the major dioxobilin‐type NCC (DNCC) in *A. thaliana* 11, 12 (see Scheme 1).

**Scheme 1:**
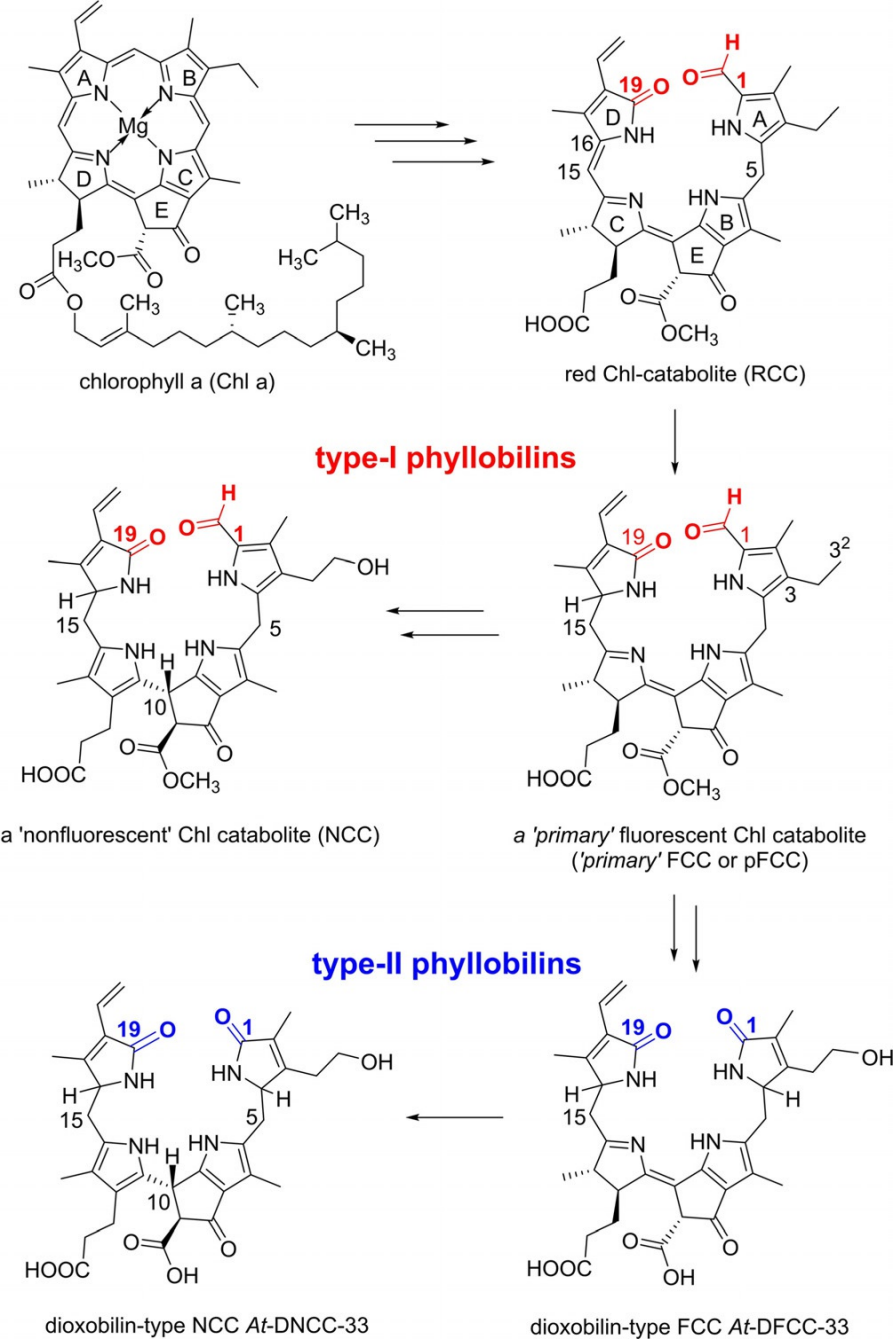
Structural outline of key steps of Chl breakdown in higher plants, furnishing two major types of colorless nonfluorescent phyllobilins (PBs), 1‐formyl‐19‐oxobilin‐type (type‐I) or 1,19‐dioxobilin‐type (type‐II) PBs.

DNCCs, named “urobilinogenoidic” Chl catabolites originally,13a were the first dioxobilin‐type PBs that hinted at a second important path of Chl breakdown,13b and DNCCs were eventually detected in a variety of senescent leaves and fruit.^5^, ^11^, ^13^ Hence, two major lines of colorless, “nonfluorescent” Chl catabolites are known to accumulate in leaves of angiosperms, representing NCCs1c, 14 or DNCCs.^5^, ^11^, 13a NCCs become oxidized in some leaves (and leaf extracts), yielding yellow Chl catabolites (YCCs), classified as type‐I phylloxanthobilins.^15^ YCCs have been detected in cold leaf and fruit extracts, in which adventitious in vitro NCC oxidation was prevented.15a, 17 By using a homogenate of a *Spatiphyllum wallisii* leaf in a “green” oxidation protocol15b, 16 YCC **2** was prepared efficiently from the epimeric NCCs **1** or **1‐*epi*** (Scheme 2). YCCs (and the analogous *py*YCCs, which lack the ring E methyl ester function) were found to self‐assemble reversibly to unique H‐bonded and π‐stacked homodimers in medium to low polarity solvents and to display exceptional medium‐dependent photochemistry.^16^, ^18^

**Scheme 2:**
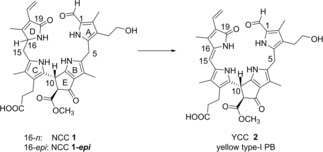
The yellow Chl catabolite YCC **2** is accessible by oxidation of both of the C16 epimeric NCCs, **1** or **1‐*epi***.

The discovery of the colorless DNCCs,13a such as of *Vv*‐DNCC‐51 (**3**),^19^ as well as their wide occurrence in senescent leaves,^5^, ^11^ in degreening vegetables13d and in fruit,13b, 20 has encouraged us to give specific attention to the presumed existence and chemical properties of dioxobilin‐type yellow PBs (DYCCs). We have provisionally detected such type‐II phylloxanthobilins recently in extracts of some naturally senescent leaves, such as *Vv*‐DYCC‐63 in leaves of grapevine (*Vitis vinifera*).^19^ Here, we describe the partial synthesis of the amphiphilic yellow type‐II PB DYCC **4** by oxidation of *Vv*‐DNCC‐51 (**3**), the major DNCC in senescent leaves of grapevine (Scheme 3), the spectral characterization of **4** and its identification with *Vv*‐DYCC‐63.

**Scheme 3:**
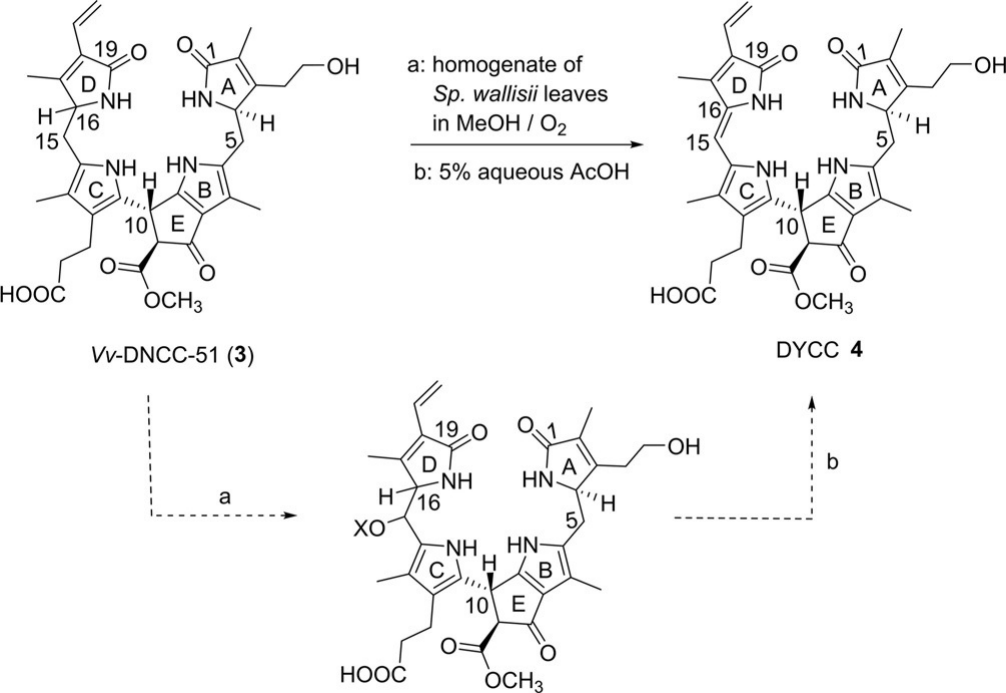
Preparation of DYCC **4** by “green” oxidation of *Vv*‐DNCC‐51 (**3**; see Ref. 19), using a homogenized senescent *Sp. wallisii* leaf, followed by acid treatment of the intermediate reaction mixture that contained polar DNCC‐like compounds (X=H and/or CH_3_). The indicated *R* configuration at C4 is from a tentative assignment for **3** and, thus, is also tentative for **4** and for its polar precursor.

For the partial synthesis of the yellow DYCC **4**, the recently described “oxidative activity” of homogenates of *S. wallisii* leaves (which oxidized some NCCs to YCCs),15b, 16 was tested here for its capacity to similarly oxidize a DNCC for preparative purposes. The abundant *Vv*‐DNCC‐51 (**3**), isolated from senescent leaves of grapevine (*V. vinifera*),^19^, ^21^ was used as the starting material (Scheme 3). A solution of *Vv*‐DNCC‐51 (5.2 mg, 8.2 μmol) in 5 mL of a 1.5:1 mixture of MeOH and aqueous phosphate buffer pH 5.2 was treated with a freshly ground yellow‐green *S. wallisii* leaf (25 cm^2^ area). The suspension was stirred for 46 hours at 23 °C under O_2_ in the dark. HPLC analysis of the filtered reaction mixture revealed significant conversion of **3** to more polar colorless DNCCs, presumed to represent the oxidized 15‐HO‐ and 15‐MeO‐derivatives of **3** (see Scheme 3). The filtrate was diluted with an aqueous 5.5 % (*v*/*v*) solution of AcOH to induce acid‐induced elimination and to generate (after 4 h reaction at room temperature) a new yellow main product, identified as DYCC **4** (see Figure S1, Supporting Information). Workup of the reaction mixture (see Experimental Section) furnished 3.6 mg (5.8 μmol, 70 % yield) of yellow DYCC **4**, which was characterized spectroscopically.

Figure 1 displays the absorption spectra of YCC **2** and of DYCC **4** in MeOH. In both spectra a strong absorption with a maximum near 428 nm was observed, indicating the presence of the same long‐wavelength absorbing chromophore, previously found in the YCC **2** 15a and in the better known yellow bile component bilirubin.^22^ In DYCC **4**, the α‐formyl‐pyrrole of ring A of **2** is replaced by an unsaturated lactam ring, manifesting itself by a marked decrease of the absorption at 310 nm, as well as by an increase of the absorption near 243 nm. Likewise, new strong bands in the circular dichroism (CD) spectrum of **4** near 320 nm and 250 nm, suggest a Cotton effect also originating from the structural and stereochemical differences associated with ring A (Figure 1). The new asymmetric carbon C4 in the DYCC **4** is tentatively assigned as *R*, based on the recent stereochemical assignment of C4 of DNCC **3**.^19^ This asymmetric carbon center is lacking in the corresponding α‐formyl‐pyrrole unit in YCCs. The further asymmetric carbons C8^2^ and C10 of DYCC **4** are inherited from the precursor, *Vv*‐DNCC‐51 (**3**), and their configuration is likely to be 8^2^(*S*) and 10(*R*), as observed in YCC **2** 16 and independently deduced for the NCCs **1** and **1‐*epi***.^5^

**Figure 1:**
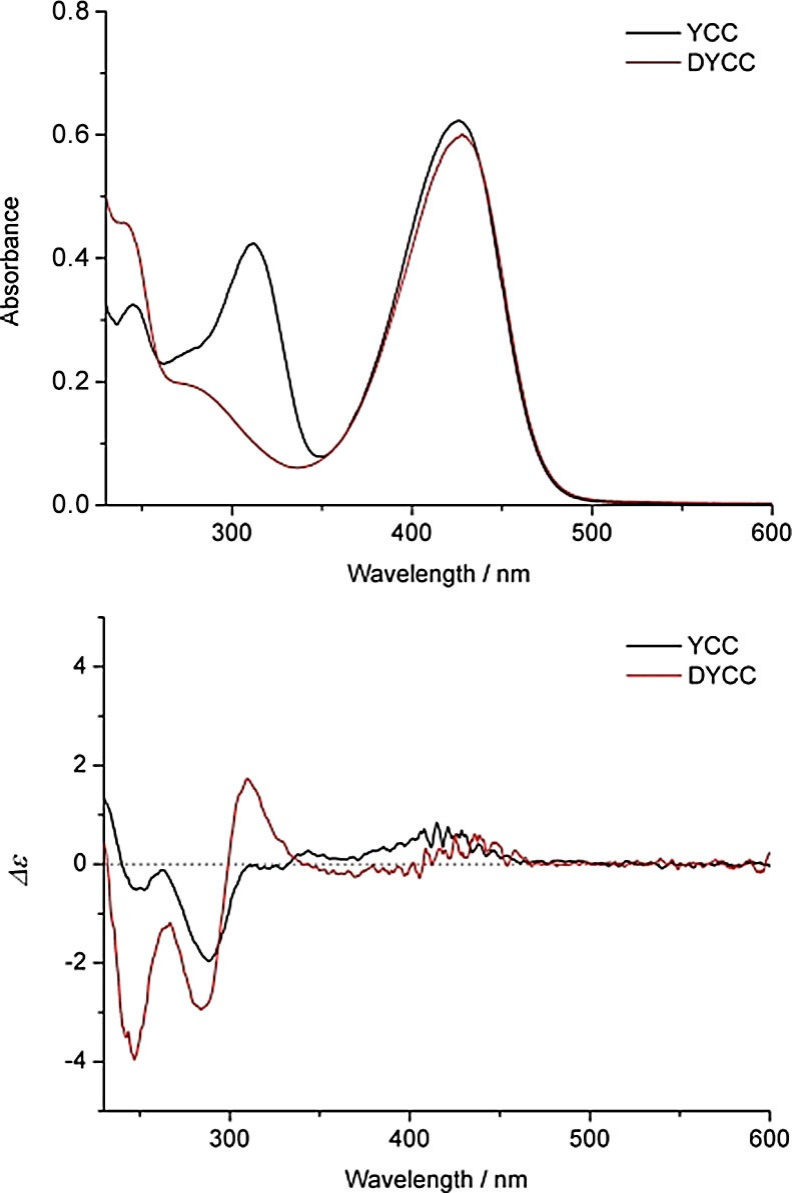
UV/Vis absorption (top) and CD (bottom) spectra of DYCC **4** (red trace) and YCC **2** (black trace) in MeOH (1.5×10^−5^ m).

The molecular formula of DYCC **4** was established as C_34_H_38_N_4_O_8_ by ESI‐MS spectrometry, and the pseudomolecular ion [*M*+H]^+^ was observed at *m*/*z=*631.1 (Figure S2a,b, Supporting Information), confirming the formal loss of two hydrogen atoms when compared to *Vv*‐DNCC‐51 (**3**).^19^ The ^1^H NMR spectrum of DYCC **4** in CD_3_OD (500 MHz, 25 °C; Figure 2), showed the signals of 31 non‐exchangeable protons, among them three signals at intermediate field with the typical pattern of a vinyl group. The structure of **4** was deduced with the help of homo‐ and heteronuclear correlation NMR spectroscopic techniques, mainly (^1^H,^1^H COSY and ROESY, ^1^H,^13^C HSQC and HMBC (Figures 2 and 3, Table S1 in the Supporting Information). A singlet at 6.22 ppm, assigned to HC15, coupled with the unsaturated C15 at 102.3 ppm, a singlet of HC10 at 5.04 ppm correlated with C10 at 37.2 ppm (HSQC correlations). The methyl ester singlet of H_3_C8^5^ at 3.77 ppm coupled to a carbonyl carbon at 171.3 ppm (HMBC). An AB spin system at 2.54 ppm and 3.09 ppm correlated with C5 at 29.9 ppm (HSQC) and was assigned to H_2_C5. It coupled with the multiplet of HC4 at 4.34 ppm in a ^1^H,^1^H COSY spectrum. Similarly, strong correlations of HC15 with the two nearby methyl groups H_3_C13^1^ and H_3_C17^1^ in a ^1^H,^1^H ROESY spectrum established the *Z* configuration at the C15=C16 bond and located the new chromophore to the left‐hand half of the molecule.

**Figure 2:**
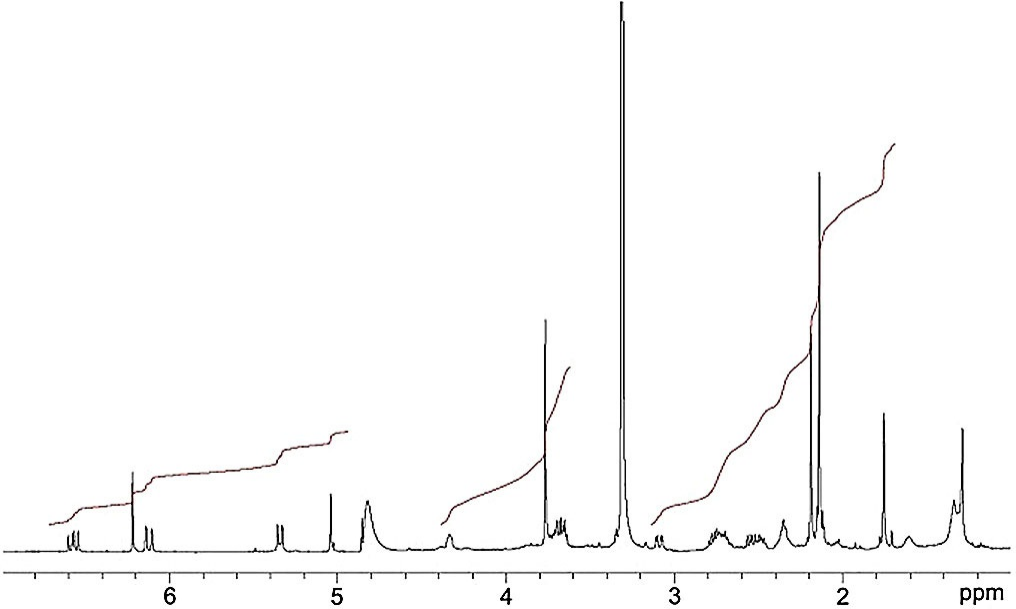
500 MHz ^1^H NMR spectrum of DYCC **4** in CD_3_OD at 25 °C.

**Figure 3:**
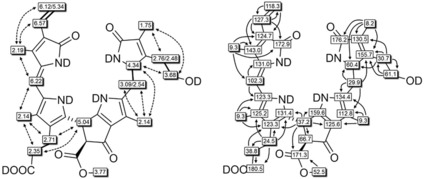
Graphical NMR‐based analysis of the structure of the DYCC **4** (data from 500 MHz spectra in CD_3_OD, 25 °C). Left: ^1^H,^1^H homonuclear correlations from COSY spectra (bold bonds) and ROESY spectra (arrows). Right: ^13^C chemical shift assignments from ^1^H,^13^C heteronuclear correlations from HSQC spectra (shaded boxes) and HMBC‐spectra (arrows/ plain boxes).

^1^H NMR spectra of **4** and **2** in CD_3_OD and their chemical shift data from homo‐ and heteronuclear correlations were compared, as was done for solutions of **4** in [D_6_]‐DMSO (Figures S3 and S4, Supporting Information). The signals of HC10, HC8^2^, the vinyl, ester methyl and three other CH_3_ groups showed comparable chemical shifts, and the signal of CHO of the formyl‐pyrrole unit of **2** was lacking in the spectrum of **4**. However, the new ring A lactam moiety of **4** generated a new multiplet at *δ*=4.34 ppm for HC4, and induced low field shifts of H_3_C2^1^, H_2_C5 and HN21. As deduced similarly from the absorption and CD spectra of **4** in MeOH or in MeCN, which display insignificant differences (Figure S5, Supporting Information), evidence for formation of H‐bonded dimers by intermolecular interaction, as seen for **2**, was not observable for solutions of **4** in these two solvents. The preparation of DYCC **4** by oxidation with DDQ, an original method used for the synthesis of YCC **2** from NCC **1**,15a was also explored here. However, under these experimentally demanding conditions, the conversion of **3** was incomplete (about 60 %) and the yield of **4** was low (22 % based on **3** converted) and the procedure was not optimized further (see Supporting Information for details).

Irradiation of a solution of 1.76 mg DYCC **4** (**4*Z***) in 50 mL MeOH in a round bottom glass flask at room temperature with sunlight led to the formation of a second, more polar DYCC fraction (HPLC analysis), the 15*E*‐isomer **4*E*** (Figure 4). Further irradiation with sun light (up to 255 min) did not significantly change the composition of the photolysis mixture that contained the two isomeric phylloxanthobilins in a molar ratio of about 3:5. The new fraction was an isomer of **4** according to its mass spectrum (Figure S7, Supporting Information). According to UV/Vis absorption and NMR spectra, the photoisomer of **4** was the double bond 15*E*‐isomer **4*E***. The absorption spectrum of **4*E*** displayed similar characteristics near 430 nm as that of 15*E*‐isomer of the YCC **2** (but lacked the 1‐formyl pyrrole absorption band near 320 nm). In its NMR spectra, DYCC **4*E*** showed diagnostic chemical shift values for the ring C/D moiety, as previously observed for the 15*E*‐isomer of YCC **2**.^16^, ^23^ Furthermore, in a 600 MHz ^1^H,^1^H ROESY spectrum of **4*E*** its low field singlet of HC15 at 6.38 ppm correlated strongly with the signal of H_3_C13^1^ at 2.04 ppm (but only very weakly with H_3_C17^1^ at 1.88 ppm), also supporting *E*‐configuration of the C15=C16 double bond (see Supporting Information and Figure S4). In the dark, solutions of **4*E*** or **4*Z*** in 1:1 ratio (*v*/*v*) of MeOH/25 mm phosphate buffer (pH7) equilibrated after storage at 23 °C for 8 days to about a 1:11 molar ratio **4*E/*4*Z*** (Figure 4).

**Figure 4:**
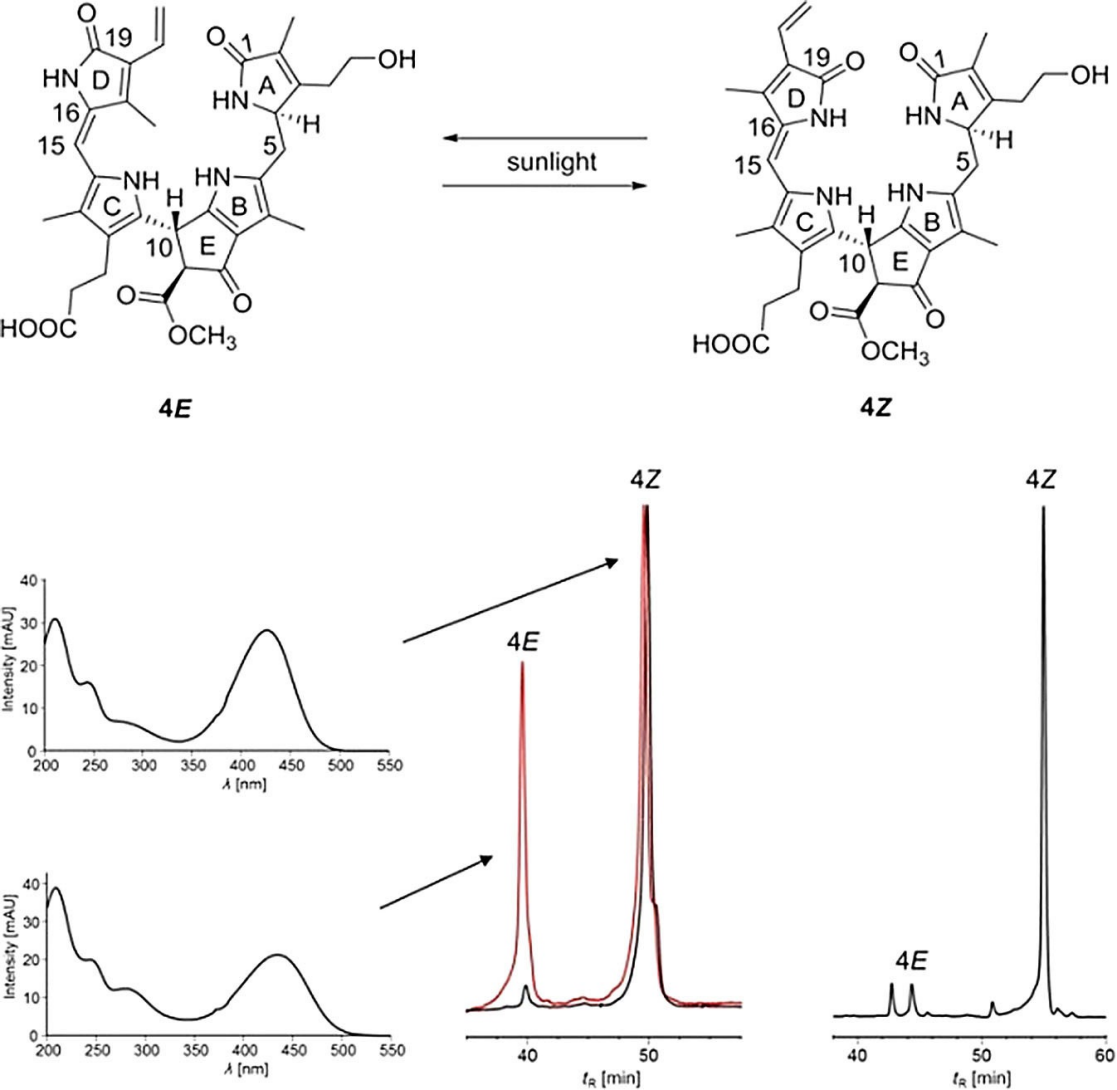
When exposed to light, the DYCC **4** (**4*Z***) isomerized to its less stable 15*E*‐isomer **4*E***, see scheme at the top. Bottom: Analyses by HPLC a fresh solution of DYCC **4** (black trace), after 25 min of irradiation with sunlight (red trace) and after storage of a solution of **4*E*** (in MeOH/25 mm phosphate buffer, pH7) for 8 days in the dark at 23 °C (right). See Supporting Information for further details. Online UV/Vis absorption spectra of **4*Z*** and **4*E*** in the photolysis mixture are depicted at the bottom, left.

In senescent leaves and fruit chlorophyll catabolites accumulate as colorless, nonfluorescent bilin‐type tetrapyrroles, primarily.2b, 5 However, yellow type‐I PBs (YCCs),15a as well as pink colored type‐I PBs (PiCCs),^23^ have also been detected in extracts of senescent leaves17b, 24 and fruit,17a as products of endogenous oxidations of NCCs or YCCs, respectively. The regioselective formation of YCC **2** from the NCCs **1** and its C16 epimer was achieved by stereo‐selective oxidation with DDQ^23^ or with leaf extracts.15b The highly regioselective outcome of this transformation was ascribed to the reactivity of the donor‐substituted pyrrole unit (ring C) of NCCs. Here, the type‐II “nonfluorescent” Chl catabolite *Vv*‐DNCC‐51,^19^ in which rings A and D have similar constitutive features, was subjected to the “leaf” oxidation protocol15b, 16, 18 and, in an exploratory experiment, to (the less productive) DDQ′ oxidation.^23^ Indeed, oxidation occurred at the left‐hand half, as expected, generating DYCC **4** with its characteristic chromophore extending over the C/d‐moiety. Hence, the structures of YCC **2** and DYCC **4** differ by the chemical constitution of ring A, only. With *Sp. wallisii* leaf homogenates as oxidation medium, evidence for the intermediate formation of polar 15‐hydroxy‐ and/or 15‐methoxy‐substituted DNCCs as direct oxidation products was provided (HPLC; see Figure S1, Supporting Information). These oxidized DNCCs underwent acid‐induced elimination of H_2_O or MeOH at C15 and C16, respectively, to furnish the yellow dioxobilin‐type Chl catabolite **4**. The “leaf” oxidation of *Vv*‐DNCC‐51 was about two‐to four times slower than the one of the NCC **1‐*epi***, making the overall preparative process somewhat slower with the DNCC, whereas the chemical yields were similar (nearly 70 %). Such oxidation reactions also occur slowly in (extracts of) naturally senescent grapevine leaves, in which *Vv*‐DYCC‐63 was detected,^19^ the structure of which was secured here by identification with DYCC **4** by HPLC (see Figure S8, Supporting Information).

Formation of a DYCC by oxidation of a DNCC (or of a YCC by oxidation of an NCC)15b desaturates the asymmetric C16 of these colorless phyllobilanes. Hence, the same YCC was obtained from C16 epimeric NCCs **1** and **1‐*epi***.15b DNCCs also occur in two C16 epimeric lines,^19^, ^25^ and C16 becomes pro‐chiral by the oxidation. However, C4 is a further asymmetric carbon center in DNCCs, not available in NCCs. The asymmetric C4 of *Vv*‐DNCC‐51 (**3**), which has tentatively been deduced to have *R* configuration,^19^ is a stereochemical result of the oxidative deformylation of an FCC‐precursor in leaves of *V. vinifera*.^19^ Consistent with a 4*R* configuration of **3**, C4 of DYCC **4** and of its *E* isomer, **4*E***, are also tentatively assigned to have *R* configuration (see Scheme 3).

YCCs undergo air oxidation, providing pink colored PiCCs,^5^, ^23^, ^26^ which are selectively accessible from YCCs via their blue zinc complex.^26^, ^27^ Although the previously studied (colored) phyllochromobilins have all represented the formyloxobilin‐type,^5^ the synthetic availability of DYCCs opens a door now to studies of the structures, photochemistry, and antioxidant effects of yellow dioxobilin‐type PBs, as well as of their capacity for metal chelation and oxidation to corresponding DPiCCs.

In contrast to the situation with the YCC **2** and its methyl ester,^16^ as well as with a *py*YCC,^18^ spectral data for DYCC **4** indicate little solvent and medium effects on spectra and structures, and no evidence for self‐assembly into dimeric structures was found for **4**. This pointed to the importance of the intact formyl‐pyrrole unit (ring A) of YCCs for their assembly to H‐bonded and π‐stacked homodimers in nonpolar solvents.^16^, ^18^ Irradiation with sunlight of a solution of the DYCC **4** in MeOH converted **4** to a new and less stable DYCC cleanly and reversibly, identified as the 15*E*‐isomer **4*E***. Hence, by undergoing selective light‐induced *E*/*Z*‐isomerization of the C15=C16 bond, the DYCC **4** exhibits the same type of photo‐reactivity as YCCs in polar solvents.

The DYCC **4** represents a new type of phylloxanthobilin (PxB),^5^ that occurs in senescent leaves (and, possibly, in fruit) and may, hence, contribute to their colors.15a, 23 In analogy to NCCs,^28^ YCCs^5^ and bilirubin^22^ DYCCs are also a group of new potential natural anti‐oxidants that may help to prolong the viability of senescent leaf tissue.^29^ Formyloxobilin‐type PxBs, such as the YCC **2**,^5^, ^27^ are unique photoswitches that feature an amazing structural similarity with some of the established heme‐derived bilins.^30^ Dioxobilin‐type PxBs, such as the DYCC **4**, are even more closely related structurally to heme‐derived bilins, such as bilirubin (BR)^22^ and the growing range of blue‐absorbing tetrapyrrolic pigments in cyanobacteriochromes.^31^ Indeed, the left‐hand half of all known PxBs carries the chromophore of bilirubin.^22^ The occurrence of type‐II PBs in leaves and fruit, and their specific and effective biosynthetic production from the first formed type‐I PBs,^11^ support interest in searching for possible biological functions of PxBs,^5^ which may relate to the roles played by some heme‐derived bilins.^22^, ^30^, ^32^

## Experimental Section

**General**: *Vv*‐DNCC‐51 (**3**)^19^ was from senescent leaves of grapevine (*Vitis vinifera*). See SI for more details.

**Spectroscopic characterization**: See Supporting Information for details.

**Preparation of DYCC (4) through “green” oxidation of Vv‐DNCC‐51 (3)**: A solution of *Vv*‐DNCC‐51 (**3**; 5.2 mg, 8.2 μmol) in 3 mL of MeOH and 2 mL of aq. phosphate buffer pH 5.2 was added to a freshly ground slurry obtained from 25 cm^2^ greenish *Sp. wallisii* leaves. The resulting mixture was stirred for 46 hours at 23 °C under 1 atm. O_2_ in the dark and then filtered through a celite pad (2 cm×1 cm). The filter cake was washed with 10 mL of MeOH. The filtrates were combined and washed with *n*‐hexane (4×10 mL) giving a light‐yellow solution that contained a polar DNCC fraction (HPLC). The combined filtrate was diluted with 30 mL of 5.5 % (pH 2.0) aqueous AcOH. After 4 hours stirring under N_2_, the reaction mixture was worked up (see Supporting Information for details), furnishing 3.6 mg (70 % yield) of **DYCC 4** as a yellow powder. UV/Vis (MeOH, *c*=1.5×10^−5^ m): *λ* (log *ϵ*) 428 (4.60), 283 (4.10), 243 nm (4.48). CD (1.5×10^−5^ m, in MeOH): *λ*_min/max_ (Δ*ϵ*): 433 (0.4), 310 (1.7), 284 (−3.0), 246 nm (−4.0); for ^1^H and ^13^C NMR data see Table S1 (Supporting Information); ESI‐MS (positive ion mode): *m*/*z* (%)*=*1285.1 (15), 1284.1 (35), 1283.1 (50; [2 *M*+Na]^+^), 1262.0 (24), 1260.9 (18; [2 *m*+H]^+^), 691.0 (23; [*M*−H+Na+K]^+^), 675.1 (14; [*M*−H+2 Na]^+^), 671.1 (13), 670.0 (25), 669.1 (64; [*M*+K]^+^),655.3 (10), 654.2 (34), 653.3 (100; [*M*+Na]^+^), 633.2 (7), 632.1 (22), 631.1 (50; [*M*+H]^+^) (see Figure S2, Supporting Information).

**Preparative photolytic isomerization of DYCC 4 (4*Z*) to its 15*E*‐isomer 4*E***: 1.76 mg (2.8 μmol) of DYCC **4** in 50 mL deoxygenated MeOH were exposed to sunlight at ambient temperature for 20 min. Work‐up and separation of the two main components furnished 0.40 μmol of **4*E*** and 1.07 μmol of **4*Z*** were recuperated (see Supporting Information for details). DYCC photoisomer **4*E***: UV/Vis (MeOH, *c*=4.9×10^−5^ m): *λ*_max_ (log *ϵ*)=206 (4.72), 246 sh (4.43), 278 (4.24), 434 nm (4.46). CD (MeOH, *c*=4.9×10^−5^ m): *λ*_max_ (Δ*ϵ*)*=*457 (2.4), 313 (5.2), 289 (−14.1), 266 (−2.6), 248 (−10.5), 227 nm (3.6); ^1^H NMR (600 MHz, CD_3_OD, 0 °C): *δ*=1.78 (s, H_3_C2^1^), 1.88 (s, H_3_C17^1^), 2.04 (s, H_3_C13^1^), 2.11 (s, H_3_C7^1^), 2.37 (t, H_2_C12^2^), 2.50 (H_A_C3^1^), 2.72 (m, H_A_C12^1^), 2.78 (m, H_B_C12^1^, H_B_C3^1^), 3.12 (AB system, *J*_AB_=4.4 Hz, H_2_C5), 3.70 (m, H_2_C3^2^), 3.76 (s, H_3_C8^5^), 4.32 (m, HC4), 4.99 (s, HC10), 5.37 (dd, *J=*2.3/11.7 Hz, H_A_C18^2^), 6.15 (dd, *J=*2.3/17.7 Hz, H_B_C18^2^), 6.38 (s, HC15), 6.54 ppm (dd, *J=*16.6/17.6 Hz, HC18^1^);^13^C NMR (150 MHz, CD_3_OD, 0 °C): *δ*=8.3 (2^1^), 9.4 (7^1^), 9.7 (13^1^), 12.5 (17^1^), 21.7 (12^1^), 30.0 (5), 30.5 (3^1^), 37.0 (10), 39.8 (12^2^), 52.6 (8^5^), 60.4 (4), 60.8 (3^2^), 67.5 (8^2^), 107.7 (15), 112.6 (7), 119.5 (18^2^), 122.1 (14), 122.4 (12), 124.4 (13), 125.5 (8), 127.3 (18^1^), 129.1 (11), 129.3 (18), 130.4 (2), 134.5 (6), 137.1 (16), 140.1 (17), 155.8 (3), 160.9 (9), 171.2 (8^3^), 171.4 (19), 176.1 (1), 181.5 ppm (12^3^); online ESI‐MS *m*/*z* (%): 685.2 (6), 669.2 (73; [*M*+K]^+^, 647.3 (7), 633.3 (6), 632.3 (36), 631.3 (95; C_34_H_39_N_4_O_8_^+^, [*M*+H]^+^), 601.3 (5), 600.3 (36), 599.3 (100; [*M*−CH_3_OH+H]^+^), 566.4 (6), 490.2 (11; [*M*−C_7_H_11_NO_2_+H]^+^), 458.2 (24; [*M*−CH_3_OH−C_7_H_11_NO_2_+H]^+^).

## Conflict of interest

The authors declare no conflict of interest.

## Supporting information

As a service to our authors and readers, this journal provides supporting information supplied by the authors. Such materials are peer reviewed and may be re‐organized for online delivery, but are not copy‐edited or typeset. Technical support issues arising from supporting information (other than missing files) should be addressed to the authors.

Supplementary
