# Supplementary material for: Yellow Dioxobilin‐Type Tetrapyrroles from Chlorophyll Breakdown in Higher Plants—A New Class of Colored Phyllobilins
Source: Chemistry. 2019 Feb 19;25(16):4052–7. doi: 10.1002/chem.201806038 (PMC6563717; doi:10.1002/chem.201806038)
Supplement: Supplementary file 1 — Supplementary [file CHEM-25-4052-s001.pdf]

CHEMISTRY
A European Journal

Supporting Information

Yellow Dioxobilin-Type Tetrapyrroles from Chlorophyll Breakdown in Higher Plants—A New Class of Colored Phyllobilins

Chengjie Li,[a, b] Theresia Erhart,[a] Xiujun Liu,[a, c] and Bernhard Kräutler*[a]

chem_201806038_sm_miscellaneous_information.pdf

Yellow Dioxobilins from Breakdown of Chlorophyll - A New Class of Colored Phyllobilins in Plants

Supporting Information

Chengjie Li, Theresia Erhart, Xiujun Liu, and Bernhard Kräutler

General. MeOH (HPLC grade), from VMR (Leuven, Belgium); water, from Millipore S. A. S. Milli-Q Academic system (18.2 MΩ·cm, Molsheim, France); ACS reagent KH_2PO_4 and K_2HPO_4, acetic acid (AcOH) from Sigma-Aldrich (Steinheim, Germany). Sep-Pak-C18 Cartridges, (Silica-based bonded phase with strong hydrophobicity), from Waters Associates (Milford, USA). pH-Values, measured with a WTW Sentix 21 electrode, WTW pH535 digital pH meter.

Spectroscopy. UV/Vis: Varian Cary 60 spectrophotometer; λ_{max} in nm (log ε). CD-spectra: JASCO J-715 spectropolarimeter; λ_{max} and λ_{min} in nm ($\Delta\varepsilon$). ^{1}H-NMR: Varian UNITY plus 500; δ in ppm with δ(CHD_2OD) = 3.31 ppm, δ(CD_3SOCD_2H) = 2.50 ppm, coupling constant J_{HH} in Hz; ^{13}C-NMR: chemical shift values and signal assignments from ^{1}H,^{13}C-HSQC and ^{1}H,^{13}C-HMBC spectra. ESI-MS (*m/z* (rel. intensity %)): Finnigan LCQ Classic, ESI source, positive ion-mode, flow rate 2 mL min^{-1}, solvent water/MeOH.

HPLC methods. *Analytical HPLC*: *Gynkotek 480G* 'high precision pump' with vacuum on-line degasser; *Gynkotek DA340* diode array detector; Column: *Phenomenex ODS-Hypersil* (5 µ, 250 × 4.6 mm i.d.) protected with pre-column; flow rate: 0.5 ml/min; all chromatograms were taken at room temperature (23 ^{o}C); solvent A: 50 mM aq. potassium phosphate (pH 7.0), solvent B: MeOH, solvent C: H_2O; solvent composition (A/B/C): 0-5 min: 60/40/0; 5-15 min: from 60/40/0 to 30/70/0; 15-25 min: from 30/70/0 to 0/100/0; 25-35 min: 0/100/0; 35-37 min: 0/100/0 to 0/90/10; 37-42 min: from 0/90/10 to 60/40/0. *Semipreparative HPLC*: Dionex

UltiMate 3000 HPLC system, UltiMate 3000 pump, UltiMate 3000 diode array detector and RF2000 fluorescence detector, 200 µl injection loop. Phenomenex Hyperclone ODS 5 µm 250 x 4.6 mm i.d. column protected with a Phenomenex ODS 4x3 mm i.d. pre-column. Data were collected and processed with Chromeleon V6.80.

Preparation of DYCC 4 from Vv-DNCC-51 by oxidation with leaf homogenate. A solution of *Vv*-DNCC-51 (**3**, 5.2 mg, 8.2 µmol) in 3 mL of MeOH and 2 mL of aq. phosphate buffer pH 5.2 was added to a freshly ground slurry obtained from 25 cm^2 greenish *Sp. wallisii* leaves. The resulting mixture was stirred for 46 hours at 23 °C under 1 atm. O_2 in the dark and then filtered through a cellite pad (2 cm × 1 cm). The filter cake was washed with 10 mL of MeOH. The filtrates were combined and washed with *n*-hexane (4 × 10 mL) giving a light yellow solution that contained a polar DNCC fraction (HPLC, see SI, Figure S1). The combined filtrate was diluted with 30 mL of 5.5 % (pH 2.0) aqueous AcOH. After 4 hours stirring under N_2, HPLC-analysis indicated full conversion to a DYCC (see SI, Figure S1) and the reaction mixture was loaded on Sep-Pak cartridge (5 g) and eluted with MeOH / potassium phosphate buffer (pH 7.0, 50 mM) (55 / 45, v / v). The obtained yellow fractions were combined and diluted with H_2O. The yellow solution was loaded on Sep-Pak cartridge (0.82 g), washed with 100 mL H_2O and the yellow product fraction was eluted with MeOH. Solvents were removed under reduced pressure and the residual material was lyophilized, furnishing a yellow powder, of 3.6 mg (70% yield) **DYCC 4** (= **4*Z***), characterized as follows: UV/Vis: (4.5 × 10^{-4} M, in MeOH) λ in nm (log ε) 428 (4.60), 283 (4.10), 243 (4.48). CD: (4.5 × 10^{-4} M, in MeOH) $\lambda_{min/max}$, nm ($\Delta\varepsilon$): 433 (0.4), 310 (1.7), 284 (-3.0), 246 (-4.0). ^{1}H-NMR (500 MHz, CD_3OD, 25 °C): δ = 1.75 (s, H_3C2^1), 2.14 (s, H_3C7^1), 2.14 (s, H_3C13^1), 2.19 (s, H_3C17^1), 2.35 (m, H_2C12^2), 2.48 (m, H_AC3^1), 2.54 (dd, *J*= 8.7, 14.8, H_AC5), 2.71 (m, H_2C12^1), 2.76 (m, H_BC3^1), 3.09 (dd, *J*=4.5, 14.8, H_AC5), 3.68 (m, H_2C3^2), 3.77 (s, H_3C8^5), 4.34 (m, HC4), 5.04 (s, HC10), 5.34 (dd, *J* = 2.0 / 11.7, H_AC18^2), 6.12 (dd, *J* = 2.0 / 17.7, H_BC18^2), 6.22 (s, HC15), 6.57 (dd, *J* = 11.7 / 17.7, $HC18^1$). ^{13}C-NMR (125 MHz, CD_3OD, 25 °C): see Table S1. ESI-MS (+-ion mode): *m*/*z* = 1320.9 (20,

$[2M–H+K+Na]^+$), 1301.1 (28), 1300.0, 1299.0 (45, $[2M+K]^+$), 1285.1 (15), 1284.1 (35), 1283.1 (50, $[2M+Na]^+$), 1262.0 (24), 1260.9 (18, $[2M+H]^+$), 691.0 (23, $[M–H+Na+K]^+$), 675.1 (14, $[M–H+2Na]^+$), 671.1 (13), 670.0 (25), 669.1 (64, $[M+K]^+$), 655.3 (10), 654.2 (34), 653.3 (100, $[M+Na]^+$), 633.2 (7), 632.1 (22), 631.1 (50, $C_{34}H_{39}N_4O_8^+$, $[M+H]^+$) (see SI, Figure S2).

Preparation of DYCC 4 from Vv-DNCC-51 by DDQ oxidation. *Vv*-DNCC-51 (**3**, 5.15 mg, 8.1 µmol) was dissolved in 0.5 mL of deoxygenated acetone. The solution was cooled down to -70 °C and 4.6 µL of AcOH was added into the solution. Cold solution of DDQ (2.03 mg, 8.9 µmol. 1.1 eq vs *Vv*-DNCC-51) in 0.25 mL of acetone was slowly added to the above solution over 20 minutes. The resulting solution was kept under Ar and allowed to warm up about -50 °C over 2 h. NaOAc (20 mg) was added to the solution and stirred at -50 °C for further 30 minutes. The mixture was diluted with 25 mL of potassium buffer (50 mM, pH 7) and loaded on a Sep-Pak cartridge (5 g). The DDQ was removed by washing with H_2O, and then with MeOH / pH 7 buffer (10 / 90, v/v). After further washing the Sep-Pak with H_2O (30 mL), the intermediate was washed down with MeOH (10 mL) and dried under reduced pressure. The obtained residue was dissolved in MeOH (1 mL) and treated with trifluoroacetic acid (TFA, 0.3 mL) for 4 minutes. The potassium buffer (50 mM, pH 7) was added to quench the reaction. The reaction mixture was loaded on Sep-Pak cartridge (5 g) and eluted with MeOH / 50 mM potassium phosphate buffer (pH 7.0) (from 50 / 50 to). *Vv*-DNCC-51 was washed down by MeOH / pH 7 buffer (50 / 50, v/v). The DYCC fraction was eluted by MeOH / pH 7 buffer (from 55 / 45 to 60 / 40, v/v), a pink fraction eluted with MeOH / pH 7 buffer (from 70 / 30 to 80 / 20, v/v). After applying to a Sep-Pak cartridge (0.82 g), the three PB fractions were first desalted with water and eluted by MeOH, respectively. After drying under reduced pressure, 2 mg of *Vv*-DNCC-51 (**3**) were re-isolated, 0.7 mg of DYCC **4** (14% yield, a yellow powder, 22% yield based on *Vv*-DNCC-51 consumed) and about 1 mg of the pink fraction were obtained.

***Additional spectral data of DYCC* 4**. UV/Vis: (2.5 × 10^{-4} M, in MeCN) λ in nm (log ε) 424 (4.58), 277 (4.15), 215 (4.49). CD: (2.5 × 10^{-4} M, in MeCN) $\lambda_{min/max}$, nm ($\Delta\varepsilon$): 433 (1.23), 311 (1.97), 283 (-3.3), 246 (-5.37). ^{1}H-NMR (500 MHz, DMSO-d_6, 25 °C): δ = 1.62 (s, H_3C2^1), 2.02 (s, H_3C7^1), 2.06 (s, H_3C13^1), 2.15 (s, H_3C17^1), 2.18 (m, H_2C12^2), 2.27 (dd, *J*= 8.9, 14.7, H_AC5), 2.32 (m, H_AC3^1), 2.56 (m, H_AC12^1), 2.60 (m, H_BC3^1), 2.62 (m, H_BC12^1), 2.95 (dd, *J*=3.6, 14.7, H_AC5), 3.47 (m, H_AC3^2), 3.53 (m, H_AC3^2), 3.66 (s, H_3C8^5), 4.14 (m, HC4), 4.18 (d, *J* = 3.9, $HC8^2$), 4.79 (d, *J* = 3.9, HC10), 5.29 (dd, *J* = 2.6 / 11.5, H_AC18^2), 6.07 (s, HC15), 6.19 (dd, *J* = 2.6 / 17.4, H_BC18^2), 6.56 (dd, *J* = 11.5 / 17.4, $HC18^1$), 8.51 (s, HN21), 9.99 (s, HN23), 10.07 (s, HN24), 13.21 (br. s, COOH). ^{13}C-NMR (125 MHz, DMSO-d_6, 25 °C): see Table S1.

Control experiment: Preparation of YCC 2 from NCC 1-epi with Sp. wallisii leaves ('green' oxidation). 10 mg (15.5 µmol) of NCC **1-*epi*** was dissolved in a mixture of MeOH / pH 5.2 K-buffer (3 mL / 3 mL). A yellow *Sp. wallisii* leaf (25 cm^2, 0.46 g) was ground with a small amount of sea sand in a mortar and the leaf slurry was added into the solution of **1-*epi***. After 6 hours at room temperature, more than 90 % (from HPLC) of NCC **1-*epi*** was oxidized to a mixture of the intermediate 15-MeO and 15-HO-adducts (HPLC analysis). Then the reaction mixture was filtered through a Celite pad (2 cm × 1 cm) and the filter cake was washed with MeOH (ca. 10 mL). The filtrate was washed with hexane until no green color in the hexane phase. The dark yellow filtrate was diluted with 5.5 % AcOH aq in H_2O (pH 2, 32 mL vs 16 mL of filtrate) and stirred at room temperature for 18 h. HPLC analysis indicted >90 % conversion to YCC **2**. The reaction mixture was loaded on Sep-Pak cartridge (5 g) and YCC **2** was washed down with 55 / 45, MeOH / 50 mM K-buffer (pH 7). After desalting on a Sep-Pak cartridge (820 mg, 1.6 mL) and lyophilization, YCC **2** was obtained as 7.2 mg (72% yield) of a yellow powder, identified by HPLC analysis, UV/Vis- and ^{1}H-NMR spectroscopy (see [Moser *et al*, *Photochem. Photobiol. Sci.* **2008**, 7, 1577]). ^{1}H-NMR (500 MHz, DMSO-d_6, 25 °C): 2.05 (s, H_3C7^1), 2.06 (s, H_3C13^1), 2.09 (m, H_AC12^2), 2.16 (s, H_3C17^1), 2.18 (s, H_3C2^1), 2.23 (m, H_BC12^2), 2.48 (m, H_2C3^1), 2.49 (m, H_2C12^1, superimposed by the signal of DMSO), 3.30 (m, H_2C3^2, superimposed by the signal of H_2O), 3.67 (s, H_3C8^5), 3.85 / 3.76 (AB system, *J* = 16.1, H_2C5) 3.87 (d, *J* = 3.7 Hz, $HC8^2$), 4.62 (br. s, OH), 4.83 (d, *J* = 3.7 Hz,

HC10), 5.32 (dd, $J = 2.5/11.5$ Hz, H_AC18^2), 6.07 (s, HC15), 6.20 (dd, $J = 2.5/17.4$ Hz, H_BC18^2), 6.57 (dd, $J = 11.5/17.4$ Hz, $HC18^1$), 9.44 (s, CHO), 10.03 (s, HN24), 10.22 (s, HN23), 11.26 (s, HN21), 11.31 (br. s, HN22), 12.04 ppm (br. s, COOH) (see Figure S3).

Photolytic isomerization of DYCC 4Z to DYCC 4**E**. A sample of 2.1 mg (2.8 µmol) of DYCC **4*Z*** was dissolved in 50 ml MeOH and the solution was flushed with argon. The yellow solution was stirred at r.t. and in the presence of sun light (3800-4800 lux, light color: 5700-6000 K) for a total of 20 min. The solvent was removed in high vacuum. The residue was dissolved in 300 µl MeOH and 600 µl H_2O and isolated by semi-preparative HPLC: flow-rate 0.5 ml min^{-1}, solvent A: 50 mM aq. potassium phosphate (pH 7.0), solvent B: MeOH; solvent composition (A/B): 0-5 min: 60/40; 5-30 min: 60/40 to 35/65; 30-35 min: 35/65 to 0/100; 35-40 min: 0/100; 40-45 min: 0/100 to 60/40. 6 consecutive HPLC runs were performed and fractions containing DYCC **4*E*** and **4*Z*** were collected. The combined DYCC **4*E*** fractions were diluted with 100 ml water and applied to a preconditioned SepPak-cartridge. After washing with 15 ml H_2O the DYCC **4*E*** was eluted with 7 ml MeOH and the solvent was removed in vacuo to give 254 µg (0.40 µmol) of DYCC **4*E***, besides 1.07 µmol of re-isolated DYCC **4*Z***.

Thermal isomerization experiments. Carefully light-protected solutions of the separated isomeric DYCCs **4*Z*** and **4*E*** in a 1 : 1 (v/v) mixture of MeOH / 50 mM aqueous phosphate buffer (pH 7) were stored at 23 ^oC for 8 days and their composition was analyzed by HPLC. Little conversion of **4*Z*** to **4*E*** was detected, but the conversion of **4*E*** to **4*Z*** was extensive (see Figure 4). The two isomers were pesent in a ratio of about 1:11 after storage of both solutions at 23 ^oC for a time of 8 d.

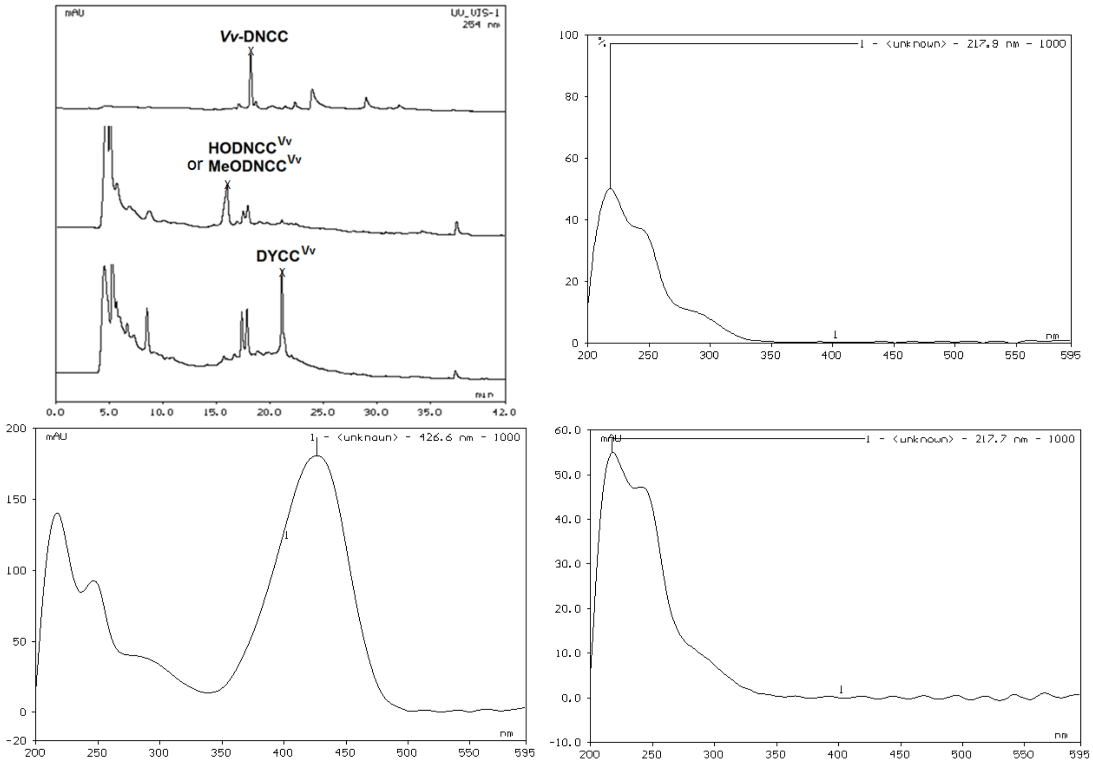

Figure S1 Left: Top. HPLC analysis of raw *Vv*-DNCC-51 in the reaction mixture of *Vv*-DNCC-51 after addition of homogenate of *Sp. wallisii* leaves, reaction mixture of *Vv*-DNCC-51 after treating with homogenate of leaves, and after treatment and reaction under acidic conditions. Bottom. online-absorption spectrum of the fraction **DYCC** (marked with “x” in the lower HPLC trace).

Right: Top. on-line absorption spectrum of *Vv*-DNCC-51 (fraction marked with “x” mark in the top HPLC trace). Bottom. on-line absorption spectrum of the fraction marked with “x” in the HPLC trace of the intermediate (tentatively identified as 15-OH and/or 15-OMe addition products of *Vv*-DNCC-51) prepared by oxidation of *Vv*-DNCC-51 with homogenate of *Sp. wallisii* leaves.

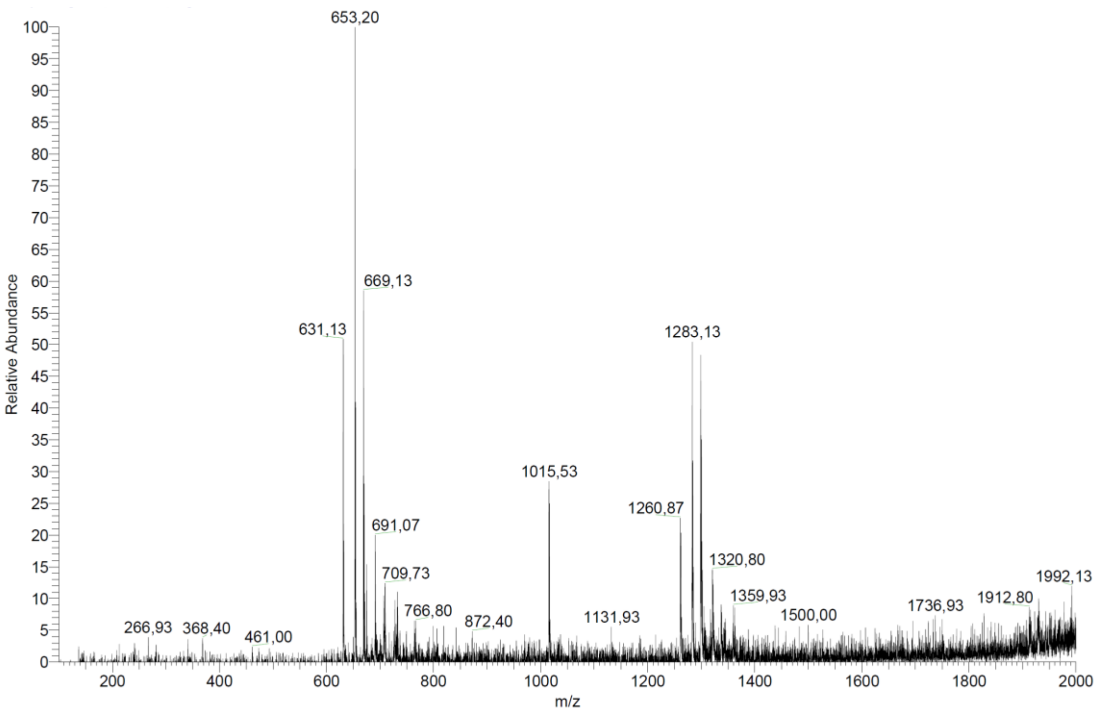

Figure S2a ESI-MS spectrum of DYCC **4** (in MeOH, ca. 2×10^{-7} M)

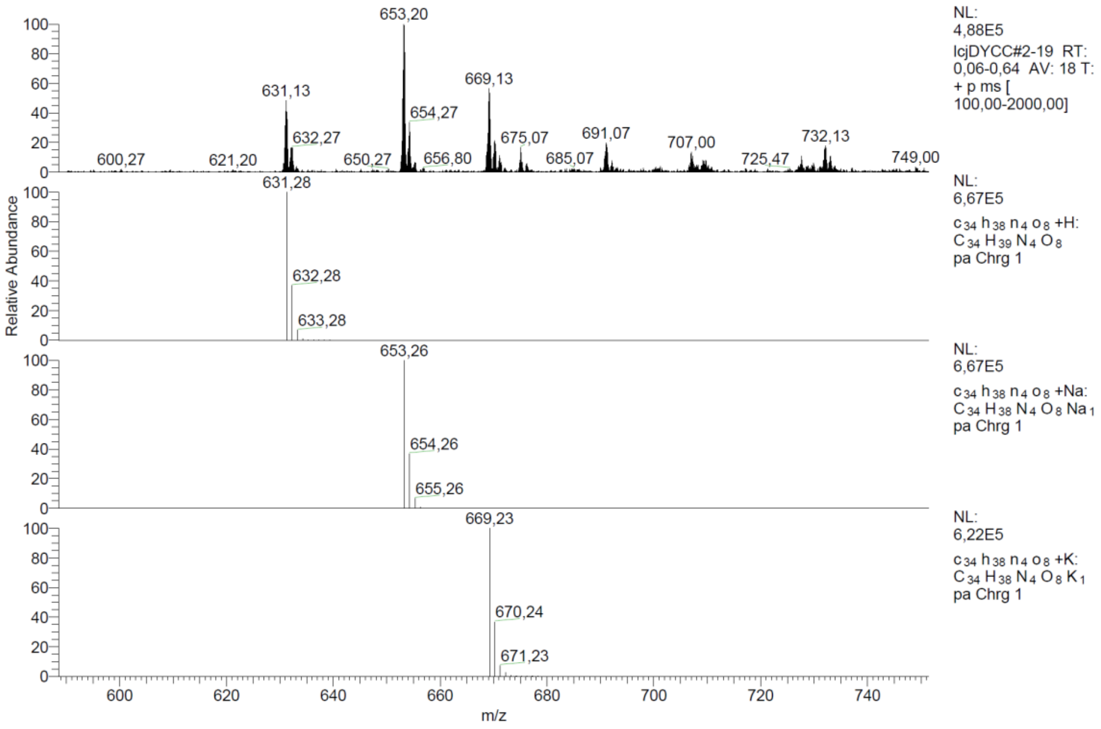

Figure S2b Simulation of the pseudo molecular ions in the lower mass range of the ESI-MS spectrum of DYCC **4**.

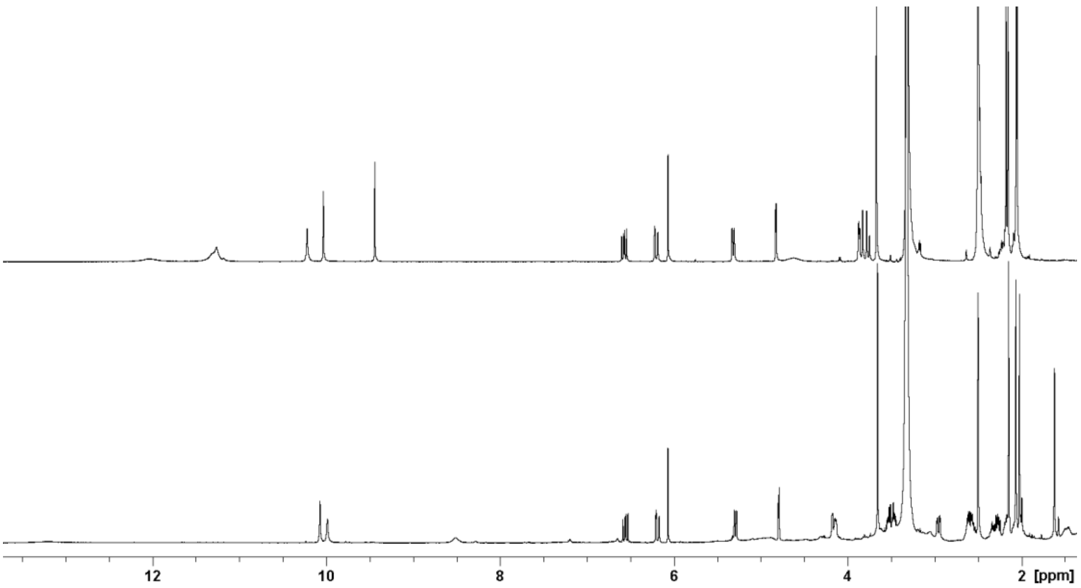

Figure S3a. 500 MHz ^{1}H-NMR spectra of YCC **2** (top) and of DYCC **4** (bottom) in DMSO-d$_6$ (25 °C).

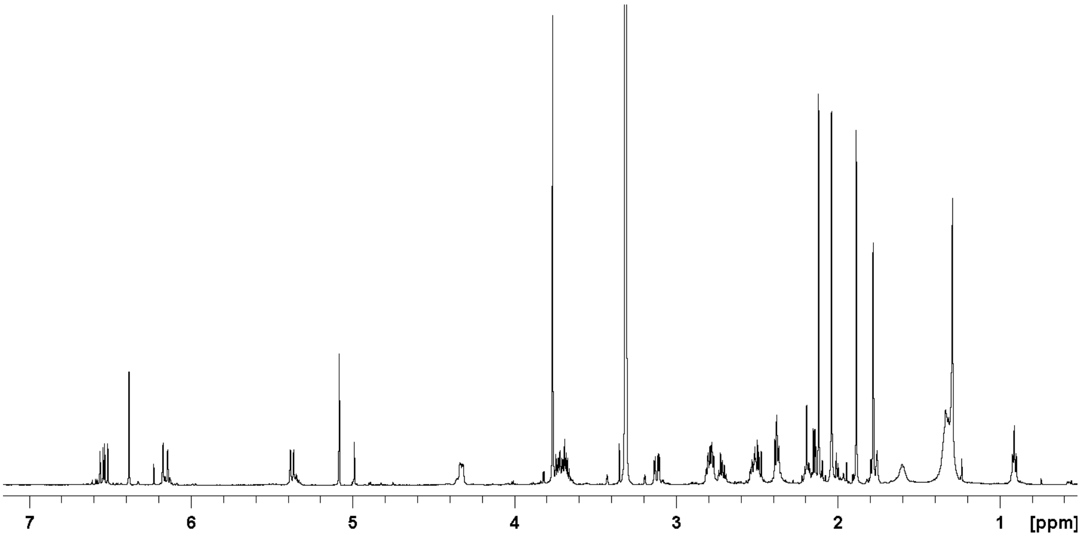

Figure S3b. 600 MHz ^{1}H-NMR spectrum of DYCC **4*E*** in CD_3OD (0 °C).

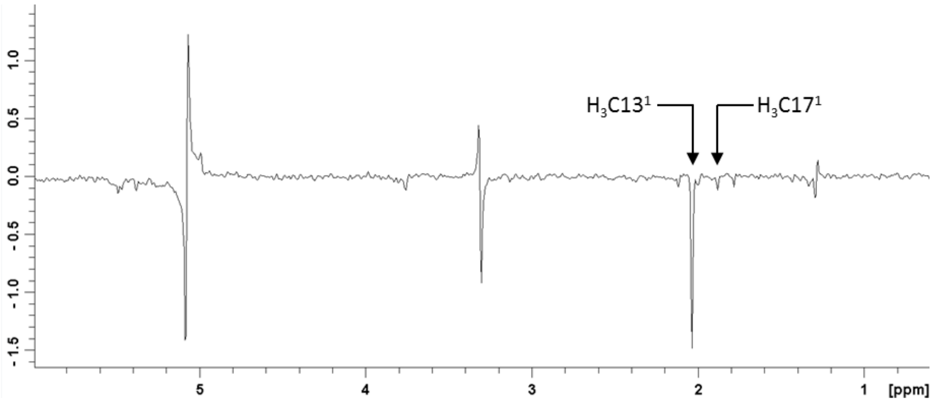

Figure S3c. Section of the ROESY-spectrum of **4E**, detected at 6.38 ppm, highlighting the correlations of HC15 with the methyl groups H_3C13^1 and H_3C17^1.

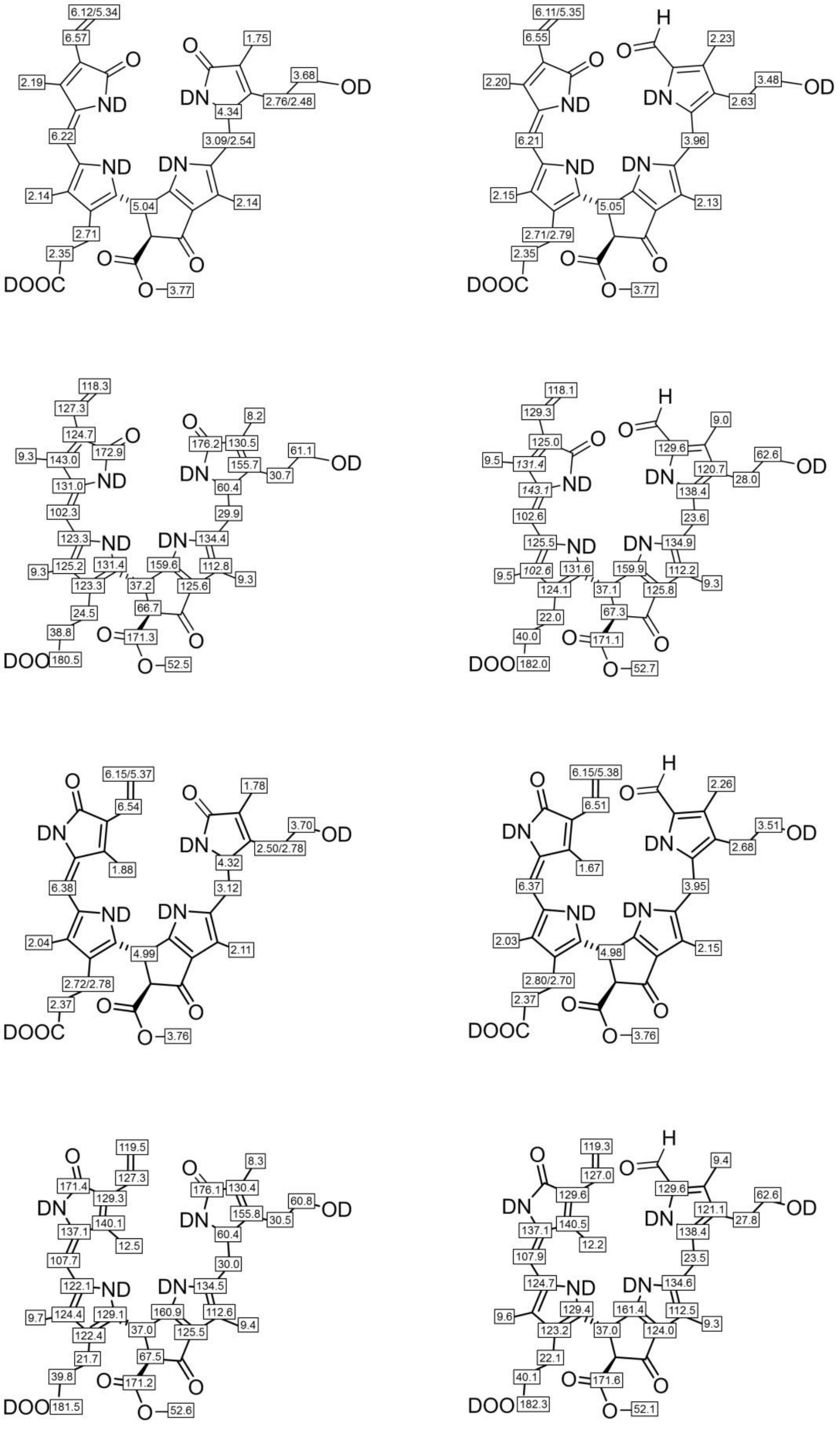

Figure S4 ^{1}H- and ^{13}C-NMR assignments (from 500 MHz spectra, CD_3OD, 25 °C) of DYCC **4*Z*** and YCC **2** (data from Moser *et al*, *Photochem. Photobiol. Sci.* **2008**, 7, 1577) and of the corresponding *E*-isomers, DYCC **4*E*** (600 MHz, 0 °C) and YCC **2*E*** (data from Ulrich *et al*, *Chem. Europ. J.* **2011**, 17, 2330; individual original assignment of C13, C16 and C17 of YCC **2**, shown in italic, were not unambiguous; ^{13}C-chemical shift values of C16 and C17 probably need to be reversed).

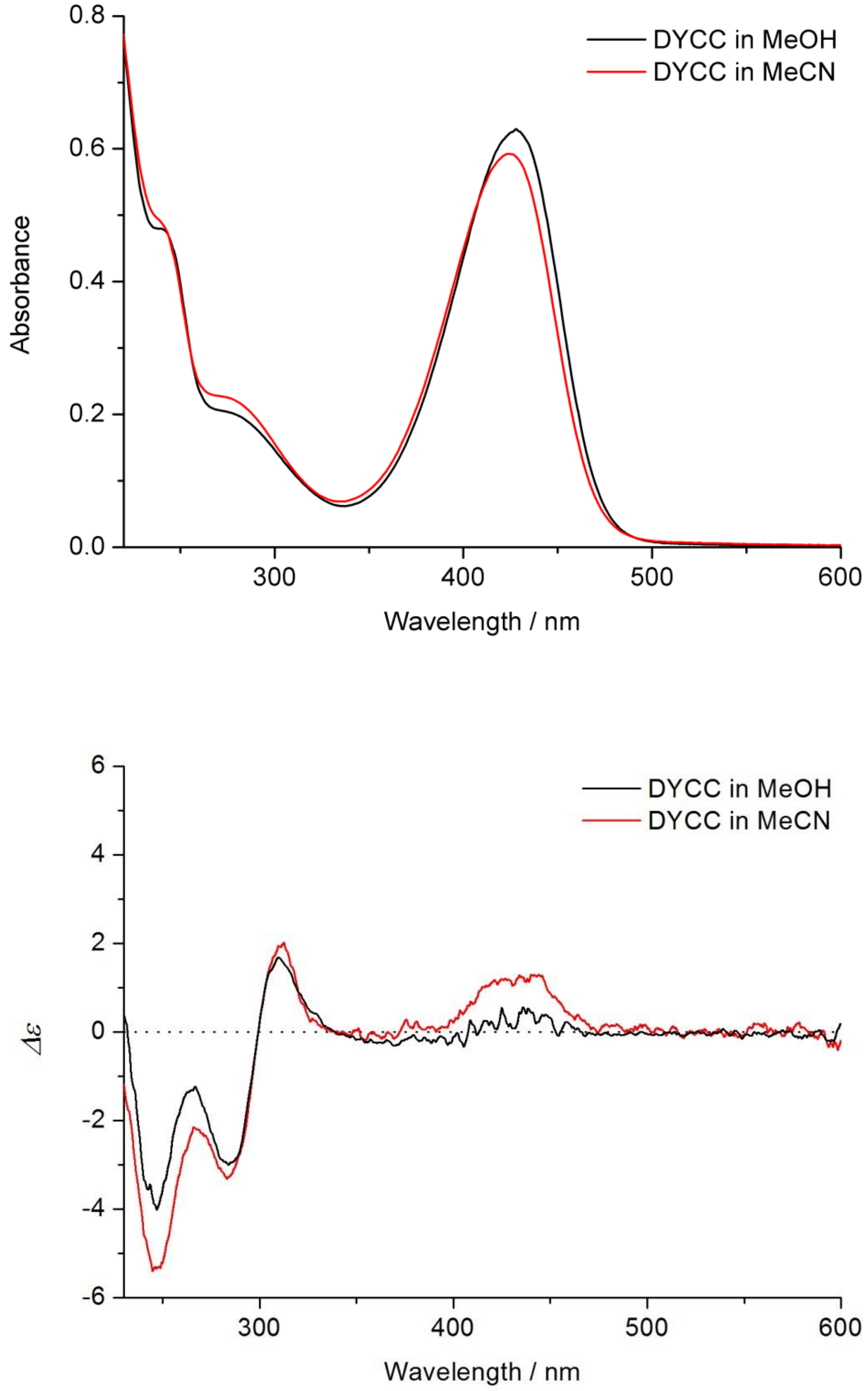

Figure S5 UV/Vis (top) and CD (bottom) spectra of DYCC **4** (= **4Z**) in MeOH and in MeCN (1.5×10^{-5} M).

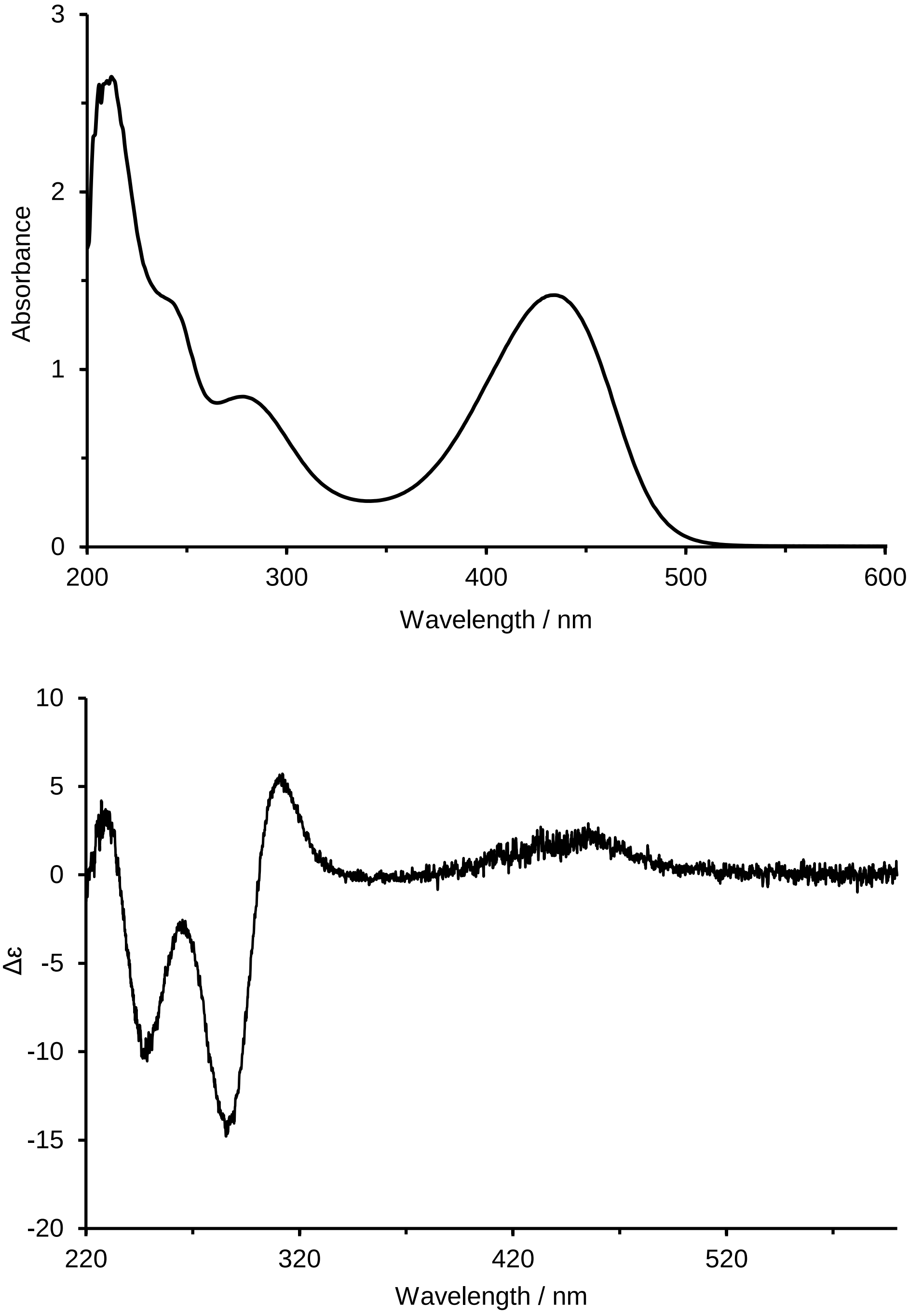

Figure S6 UV/Vis-spectrum (top) and CD-spectrum (bottom) of DYCC **4*E*** in MeOH (4.9×10^{-5} M).

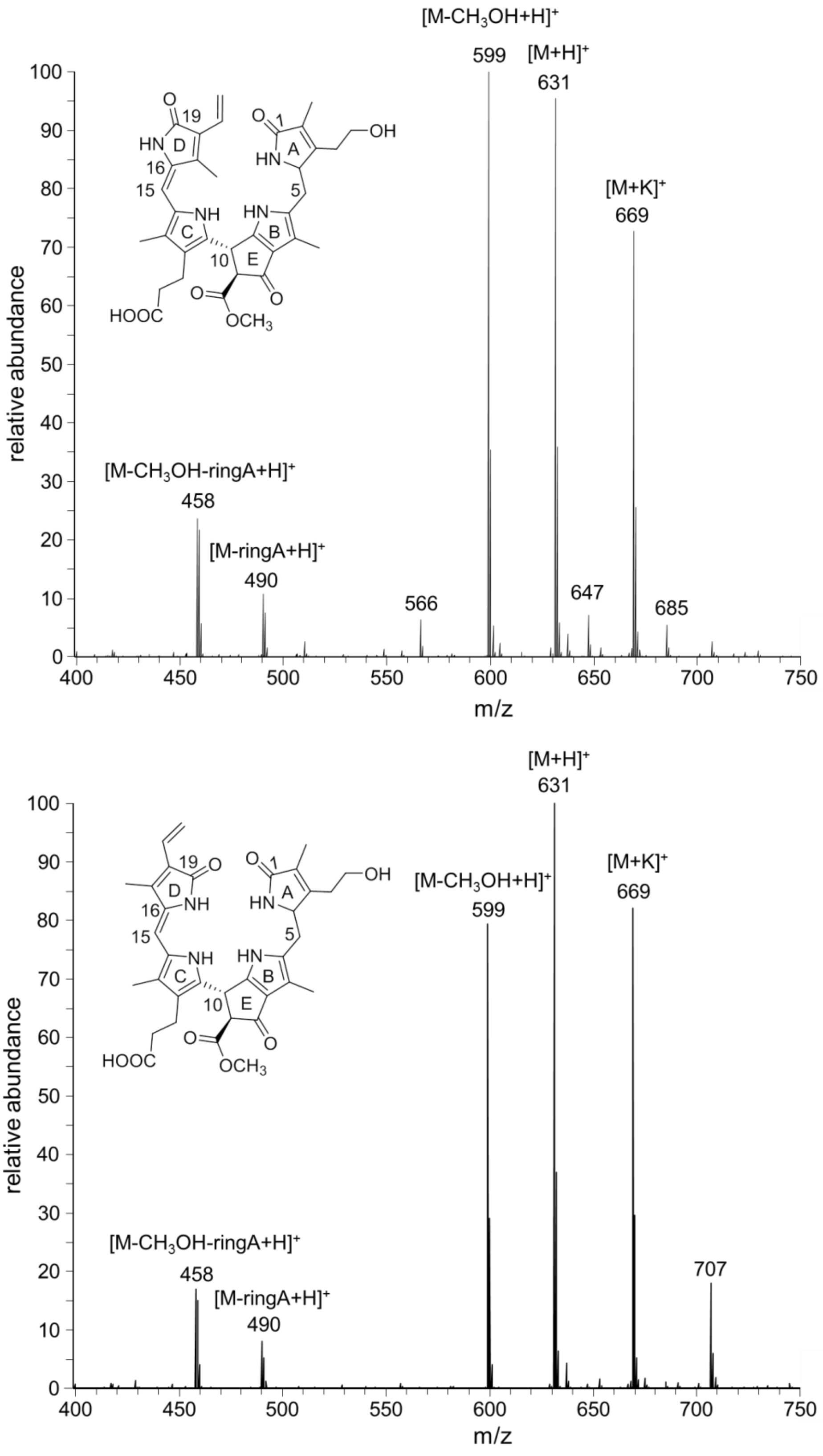

Figure S7. Section of the intermediate mass range of the ESI-MS spectra of ***4E*** (top) and ***4Z*** (bottom), depicting the pseudo-molecular ions ([M+H]$^+$ and ([M+K]$^+$) as well as fragments from loss of CH_3OH, of ring A or both.

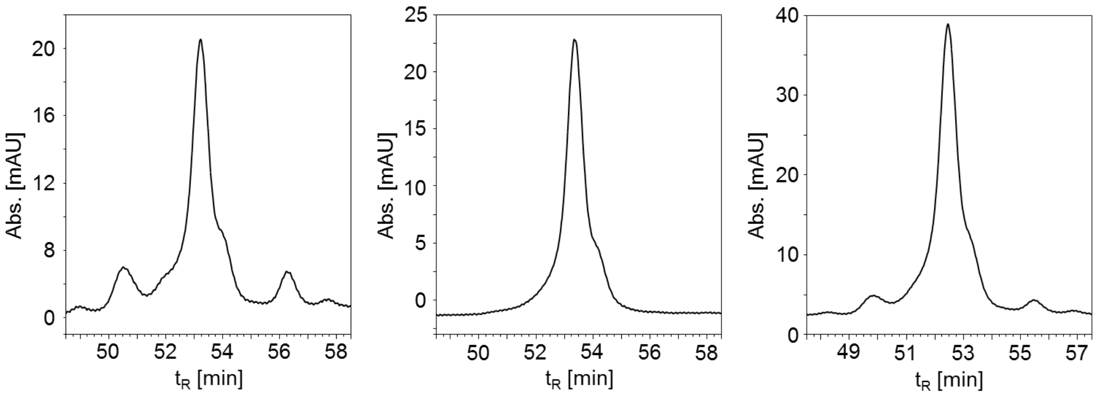

Figure S8. Section of analytical HPLC traces for elution times between 49 and 58 min of (left) an extract of a yellow, senescent Chardonnay leaf, of (center) a solution of DYCC **4*Z*** and of (right) a roughly 1:1 mixture of the leaf extract and of the solution of the DYCC **4*Z*** (detection at 420 nm).

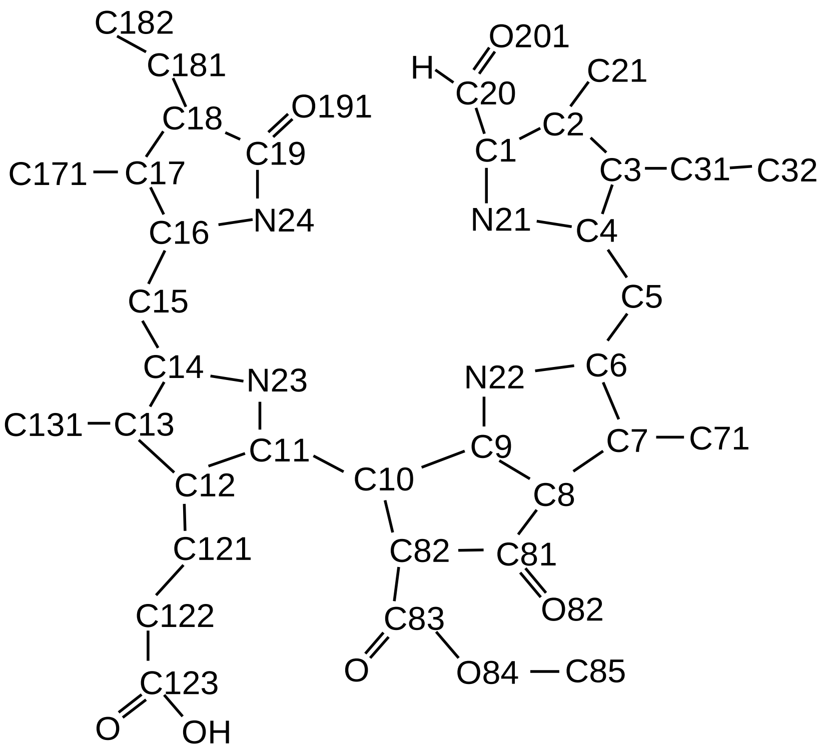

Fig. S9. Atom numbering used for type-II phyllobilins.

Table S1 ^{1}H- and ^{13}C-NMR data of DYCC **4** in DMSO-d$_6$ and in CD_3OD (500 MHz, 25 °C).

	DYCC **4** in DMSO-d$_6$		DYCC **4** in CD_3OD	
	^{1}H	^{13}C	^{1}H	^{13}C
C1		173.1		176.2
C2		128.4		130.5
H_3C2^1	1.62 (s)	8.1	1.75 (s)	8.2
C3		153.4		155.7
H_2C3^1	2.32 (m) / 2.60 (m)	29.7	2.48 (m) / 2.76 (m)	30.7
H_2C3^2	3.47 (m) / 3.53 (m)	58.8	3.68 (m)	61.1
$HO3^3$				
HC4	4.14 (d, 7.9)	58.5	4.34 (m)	60.4
H_AC5	2.27 (dd, 8.9, 14.7)	29.4	2.54 (dd, 8.7, 14.8)	29.9
H_BC5	2.95 (dd, 3.6, 14.7)		3.09 (dd, 4.5, 14.8)	
C6		133.9		134.4
C7		109.3		112.8
H_3C7^1	2.02 (s)	9.0	2.14 (s)	9.3
C8		122.9		125.6
$HC8^2$	4.18 (d, 3.9)	64.5		66.7
$C8^3$		170.2		171.3
$C8^5$		51.8	3.77 (s)	52.5
C9		158.4		159.6
C10	4.79 (d, 3.9)	35.4	5.04 (s)	37.2
C11		130.6		131.4
C12		122.2		123.3
H_2C12^1	2.56 (m) / 2.62 (m)	20.8	2.71 (m)	24.5
H_2C12^2	2.18 (m)	38.5	2.35 (m)	38.8
$C12^3$		176.2		180.5
C13		123.7		125.2
H_3C13^1	2.06 (s)	9.2	2.14 (s)	9.3
C14		122.2		123.3
HC15	6.07 (s)	99.5	6.22 (s)	102.3
C16		128.7		131.0
C17		142.0		143.0
H_3C17^1	2.15 (s)	9.0	2.19 (s)	9.3
C18		122.6		124.7
$HC18^1$	6.56 (dd, 11.5, 17.4)	126.8	6.57 (dd, 11.7, 17.7)	127.3
H_AC18^{2cis}	5.29 (dd, 2.6, 11.5)	117.1	5.34 (dd, 2.0, 11.7)	118.3
H_BC18^{2trans}	6.19 (dd, 2.6, 17.4)		6.12 (dd, 2.0, 17.7)	
C19		170.3		172.9
HN21	8.51 (s)			
HN22				
HN23	9.99 (s)			
HN24	10.07 (s)			
